# Professor Chapman

**Published:** 1848-04

**Authors:** 


### PROFESSOR CHAPMAN.

The Medical Class of the University of Pennsylvania, to testify their respect for their venerable Professor of the Theory and Practice of Medicine, Dr. N. Chapman, have had his portrait painted by Sully, in the best style of that distinguished artist. Beside being a beautiful specimen of art, it is said to be an accurate likeness. On the 22d ult. it was presented with appropriate ceremonies to the Wistar Museum of the University, to be placed by the side of those of the Professor's former colleagues—Physic and Dewees.

